# Hydrophobins from *Aspergillus *species cannot be clearly divided into two classes

**DOI:** 10.1186/1756-0500-3-344

**Published:** 2010-12-23

**Authors:** Britt G Jensen, Mikael R Andersen, Mona H Pedersen, Jens C Frisvad, Ib Søndergaard

**Affiliations:** 1 Center for Microbial Biotechnology, Department of Systems Biology, Technical University of Denmark, Søltofts Plads, Building 221/223, DK-2800 Kgs. Lyngby, Denmark

## Abstract

**Background:**

Hydrophobins are a family of small secreted proteins with a characteristic pattern of eight cysteine residues found exclusively in filamentous fungi. They have originally been divided into two classes based on their physical properties and hydropathy patterns, and are involved in the attachment of hyphae to hydrophobic structures, the formation of aerial structures and appear to be involved in pathogenicity.

**Findings:**

Analysis of nine genome sequences from seven Aspergilli revealed fifty hydrophobins, where each species displayed between two to eight hydrophobins. Twenty of the identified hydrophobins have not previously been described from these species. Apart from the cysteines, very little amino acid sequence homology was observed. Twenty-three of the identified hydrophobins could be classified as class I hydrophobins based on their conserved cysteine spacing pattern and hydropathy pattern. However twenty-six of the identified hydrophobins were intermediate forms. Notably, a single hydrophobin, ATEG_04730, from *Aspergillus terreus *displayed class II cysteine spacing and had a class II hydropathy pattern.

**Conclusion:**

Fifty hydrophobins were identified in *Aspergillus*, all containing the characteristic eight cysteine pattern. *Aspergillus terreus *exhibited both class I and class II hydrophobins. This is the first report of an *Aspergillus *species with the potential to express both class I and class II hydrophobins. Many of the identified hydrophobins could not directly be allocated to either class I or class II.

## Background

Hydrophobins are a family of small proteins found uniquely in filamentous fungi [1]. The currently characterised hydrophobins are approximately 100 AA in size and have little amino acid sequence homology except from eight conserved cysteines in a characteristic pattern [2,3]. The eight cysteines form four disulfide bonds in the pattern Cys1-Cys6, Cys2-Cys5, Cys3-Cys4, Cys7-Cys8 and especially the Cys3-Cys4 loop can vary considerably in length [4]. Based on their distinct hydropathy patterns and physical properties, hydrophobins are traditionally divided into two classes [3]. Class I hydrophobins form highly insoluble membranes in water, organic solvents and 2% SDS, while the membranes formed by class II hydrophobins easily can be dissolved in aqueous ethanol (60%) or 2% SDS [2]. Class I hydrophobins have been identified in Ascomycetes and Basiodiomycetes, while class II hydrophobins have only been identified in Ascomycetes [1]. Typically, a single fungal species only expresses either class I or class II hydrophobins, however previous studies have shown that few species have the ability to express both class I and class II hydrophobins [5,6]. In class I hydrophobins the cysteine doublets are followed by hydrophilic amino acids, while hydrophobic amino acids are observed after the cysteine doublets in class II hydrophobins [2]. Furthermore, considerable variation is seen in the cysteine spacing of class I hydrophobins, while less variation is seen for class II hydrophobins [7]. In this study, we examine nine full genome sequenced Aspergilli for new hydrophobins.

## Results and Discussion

### Identification of hydrophobins

Nine full genome sequenced *Aspergillus *species were used to search for new hydrophobins. A total of 50 potential hydrophobins were identified (Table 1) based on the criteria of minimum eight cysteines, two cysteine pairs, a size of app. 100 AA and the cysteine pattern. On species level twenty of the identified hydrophobins have not previously been mentioned in other studies, while the number increases to thirty-one on strain level. The number of identified hydrophobins within the species varied from two to eight between the nine species. All identified hydrophobins had theoretical signal sequences and therefore have the possibility of being secreted. They contain approximately 100 - 200 amino acids and are 8 - 30 kDa in size. Furthermore, they contain eight to ten cysteines, where excess cysteines (above eight) are located before or after the conserved cysteine spacing pattern. Beauvais *et al*. (2007) have classified AFUA_8G05890 and AFUA_5G01490 as hydrophobins. As AFUA_8G05890 has 11 cysteines, no signal sequence and both proteins lack the conserved cysteine pattern, we disregard these proteins as hydrophobins as they do not fulfil our criteria. Other *Aspergillus *hydrophobins previously identified fulfilled the criteria [8-16] and have likewise been found and included in this study.

**Table 1:** Aspergillus hydrophobins

Species	Gene	Size (Da)	n(AA)	n(cys)	Eight cysteine pattern	Theoretical class	Common name
*A. oryzae *RIB40	AO090012000143	14304	145	8	CN{8}CCN{38}CN{10}CN{5}CCN{21}C	I	RolA^a^
	AO090020000588	15231	151	8	CN{7}CCN{39}CN{17}CN{5}CCN{17}C	I	New
*A. niger *CBS 513.88	An03g02360^b^	12486	122	8	CN{6}CCN{32}CN{25}CN{5}CCN{4}C	I	
	An03g02400^b^	13063	131	8	CN{6}CCN{31}CN{23}CN{5}CCN{6}C	Intermediate	
	An04g08500^b^	14397	146	8	CN{7}CCN{39}CN{20}CN{5}CCN{17}C	I	
	An15g03800^b^	13225	130	8	CN{5}CCN{32}CN{6}CN{5}CCN{13}C	Intermediate	
	An01g10940^b^	10693	100	8	CN{14}CCN{17}CN{11}CN{7}CCN{8}C	Intermediate	
	An07g03340^b^	16207	162	8	CN{7}CCN{39}CN{21}CN{5}CCN{17}C	I	
	An09g05530^b^	20465	202	9	CN{8}CCN{33}CN{11}CN{5}CCN{16}C	Intermediate	
	An08g09880^b^	9169	91	9	CN{7}CCN{16}CN{6}CN{5}CCN{10}C	Intermediate	
*A. niger *ATCC 1015	JGI128530	10803	105	7	Fragment (similar to An07g03340)	Intermediate	(New)
	JGI35683	10693	100	8	CN{14}CCN{17}CN{11}CN{7}CCN{8}C	Intermediate	(New)
	JGI45683	13063	131	8	CN{6}CCN{31}CN{23}CN{5}CCN{6}C	Intermediate	(New)
	JGI45685	13716	132	8	CN{6}CCN{32}CN{25}CN{5}CCN{14}C	I	(New)
	JGI53462	13224	130	8	CN{5}CCN{32}CN{6}CN{5}CCN{13}C	Intermediate	(New)
	JGI194815	14397	146	8	CN{7}CCN{39}CN{20}CN{5}CCN{17}C	I	(New)
	JGI43184	20381	201	9	CN{8}CCN{33}CN{11}CN{5}CCN{16}C	Intermediate	(New)
*E. nidulans *FGSC A4	AN7539.2^c^	10798	109	8	CN{5}CCN{32}CN{6}CN{5}CCN{13}C	Intermediate	
	AN8803.2^c^	15625	157	8	CN{7}CCN{39}CN{18}CN{5}CCN{17}C	I	RodA^d^
	AN6401.2^c^	16131	162	8	CN{6}CCN{38}CN{22}CN{5}CCN{35}C	Intermediate	
	AN8006.2^c^	13183	135	8	CN{6}CCN{31}CN{23}CN{5}CCN{6}C	I	DewA^e^
	AN1837.2^c^	13397	135	8	CN{7}CCN{39}CN{18}CN{5}CCN{17}C	I	
	AN0940.2^c^	10594	101	8	CN{13}CCN{17}CN{12}CN{7}CCN{8}C	Intermediate	
*A. fumigatus *AF293	AFUA_8G07060	15996	155	8	CN{7}CCN{39}CN{21}CN{5}CCN{17}C	I	RodC^f^
	AFUA_5G09580	16153	159	8	CN{7}CCN{39}CN{21}CN{5}CCN{17}C	I	RodA^f/g^
	AFUA_2G14661	12928	125	8	CN{5}CCN{32}CN{6}CN{5}CCN{13}C	Intermediate	New
	AFUA_1G17250	14299	140	8	CN{7}CCN{36}CN{18}CN{5}CCN{18}C	I	RodB^f/h^
	AFUA_5G03280	19825	190	9	CN{7}CCN{33}CN{11}CN{5}CCN{14}C	I	RodF^f^
*A. fumigatus *A1163	AFUB_016640	14300	140	8	CN{7}CCN{36}CN{18}CN{5}CCN{18}C	I	(RodB New)
	AFUB_057130	16153	159	8	CN{7}CCN{39}CN{21}CN{5}CCN{17}C	I	(RodA New)
	AFUB_080740	15996	155	8	CN{7}CCN{39}CN{21}CN{5}CCN{17}C	I	(RodC New)
	AFUB_051810	19825	190	9	CN{7}CCN{33}CN{11}CN{5}CCN{14}C	Intermediate	(RodF New)
*A. terreus *NIH 2624	ATEG_10285	13978	129	8	CN{5}CCN{28}CN{14}CN{8}CCN{13}C	Intermediate	New
	ATEG_08089	18936	177	8	CN{8}CCN{33}CN{11}CN{5}CCN{14}C	Intermediate	New
	ATEG_07808	11677	115	8	CN{5}CCN{32}CN{6}CN{5}CCN{13}C	Intermediate	New
	ATEG_06492	17374	175	8	CN{7}CCN{40}CN{16}CN{5}CCN{17}C	I	New
	ATEG_04730	11797	121	8	CN{10}CCN{11}CN{16}CN{8}CCN{10}C	II	New
*A. flavus *NRRL 3357	AFLA_094600	8377	83	8	CN{7}CCN{16}CN{6}CN{5}CCN{9}C	Intermediate	New
	AFLA_131460	10867	106	8	CN{5}CCN{32}CN{6}CN{5}CCN{13}C	Intermediate	New
	AFLA_060780	27807	251	8	CN{6}CCN{30}CN{23}CN{5}CCN{4}C	I	New
	AFLA_014260	14304	145	8	CN{8}CCN{38}CN{10}CN{5}CCN{21}C	I	New
	AFLA_063080	9362	87	9	CN{5}CCN{17}CN{7}CN{7}CCN{12}C	Intermediate	New
	AFLA_098380	23415	217	10	CN{7}CCN{39}CN{17}CN{5}CCN{44}C	I	New
	AFLA_064900	9147	91	10	CN{7}CCN{15}CN{6}CN{5}CCN{8}C	Intermediate	New
*A. clavatus *NRRL 1	ACLA_001890	10214	100	8	CN{7}CCN{16}CN{6}CN{5}CCN{26}C	Intermediate	New
	ACLA_048810	18458	182	8	CN{7}CCN{33}CN{11}CN{5}CCN{15}C	Intermediate	New
	ACLA_010960	14671	145	8	CN{7}CCN{39}CN{21}CN{5}CCN{17}C	I	New
	ACLA_072820	16127	158	8	CN{7}CCN{39}CN{21}CN{5}CCN{17}C	I	New
	ACLA_018290	12820	126	8	CN{5}CCN{32}CN{6}CN{5}CCN{13}C	Intermediate	New
	ACLA_007980	14558	144	8	CN{7}CCN{36}CN{18}CN{5}CCN{17}C	Intermediate	New

^a ^described by [9],^b ^mentioned by [10], ^c ^mentioned by [11], ^d ^described by [12], ^e ^described by [8], ^f ^described by [13], ^g ^RodA described by [14,15], ^h ^RodB described by [16]

Forty-five of the identified proteins contained domains classifying them as hydrophobins by Pfam. The remaining five hydrophobins could not be classified. Four of these (An01g10940, JGI35683, AN0940.2, AFLA_063080) can be differentiated from the rest in displaying a distinctive cysteine pattern. They have a similar cysteine pattern of CN{5-13}CCN{17}CN{7-12}CN{7}CCN{8-12}C (where N signifies any other amino acid than cysteine) and group together in the phylogenetic tree (Additional file 1), but still with other hydrophobins. They also have hydropathy patterns that differ from both class I and class II hydrophobins and can therefore theoretically not be placed in either class. Furthermore, their hydropathy patterns differ from each other, so they do not form a new class either. The fifth hydrophobin (ATEG_10285) differs in having a different cysteine spacing compared to all other identified hydrophobins, but still clusters with other hydrophobins in the phylogenetic tree (Additional file 1). Forty-four of the identified hydrophobins displayed class I cysteine spacing pattern, but only twenty-four had a characteristic class I hydropathy plot resulting in only twenty-three identified class I hydrophobins (see Additional file 2 and Table 1). Only one identified hydrophobin displayed a characteristic class II cysteine spacing pattern and had a class II hydropathy pattern, while the rest (twenty-six) were intermediate forms. However, as the majority of the identified hydrophobins have not physically been isolated and characterised, a differentiation into type of class is only provisional. As many of the identified hydrophobins displayed intermediate forms, they may also exhibit solubility characteristics between the two known classes. As these intermediate forms blur the original classification, it could be speculated, whether an extension of the classical two class system would be in place as more fungal genomes become available.

An examination of the multiple alignment (Additional file 3) of the putative hydrophobins revealed very low similarity between the hydrophobins. Apart from the eight cysteines a proline was observed in the majority of the sequences (82%) situated in close proximity to the theoretical signal sequence cleavage site. This proline may be involved in the correct cleavage of the signal sequence and thereby influence the eventual secretion of the hydrophobins. Tryptophan is rarely seen in hydrophobins [2], and only twelve of the identified hydrophobins from Aspergilli contained between 1-5 tryptophan residues.

Several groups are revealed in the phylogenetic tree (Additional file 1) and it seems that hydrophobins cluster according to their cysteine spacing pattern. A common feature in 44 of the 50 hydrophobins is a conserved spacing of five amino acids between the fifth and sixth cysteines, while the remaining six hydrophobins contain either seven or eight amino acids. This spacing of five cysteines is also observed in other known class I hydrophobins (eg. SC3, EAS and MPG1) [7] and may be a common feature in class I hydrophobins.

Previously Yang *et al*. (2006) [17] used primary structure analysis to identify new members of the hydrophobin family. By searching the Uniprot Knowledgebase using the key word hydrophobin followed by a BLAST against the NCBI database, Yang *et al*. retrieved several sequences. However, by using the above mentioned method putative hydrophobin sequences may be missed as hydrophobins have high sequence diversity, and may not resemble known hydrophobins sufficiently to be picked up by a BLAST. In our search we found five hydrophobins (An01g10940, JGI35683, AN0940.2, AFLA_063080, ATEG_10285), which do not resemble the other identified hydrophobins. If these hydrophobins are used to conduct a BLAST, no known hydrophobins appear in the results. So if the method described by Yang *et al*. was used, these putative hydrophobins would likely have been missed. Furthermore, Yang *et al*. uses their identified sequences to create motifs, and thereby identify nine new hydrophobins including five *E. nidulans *(*A. nidulans*) hydrophobins. In our approach we only sort our putative hydrophobins by the criteria of size, number of cysteines and the eight cysteine pattern, thereby not eliminating any hydrophobins even if they do not contain any common motifs.

### Class I and class II hydrophobins of *Aspergillus terreus*

**Figure 1:**
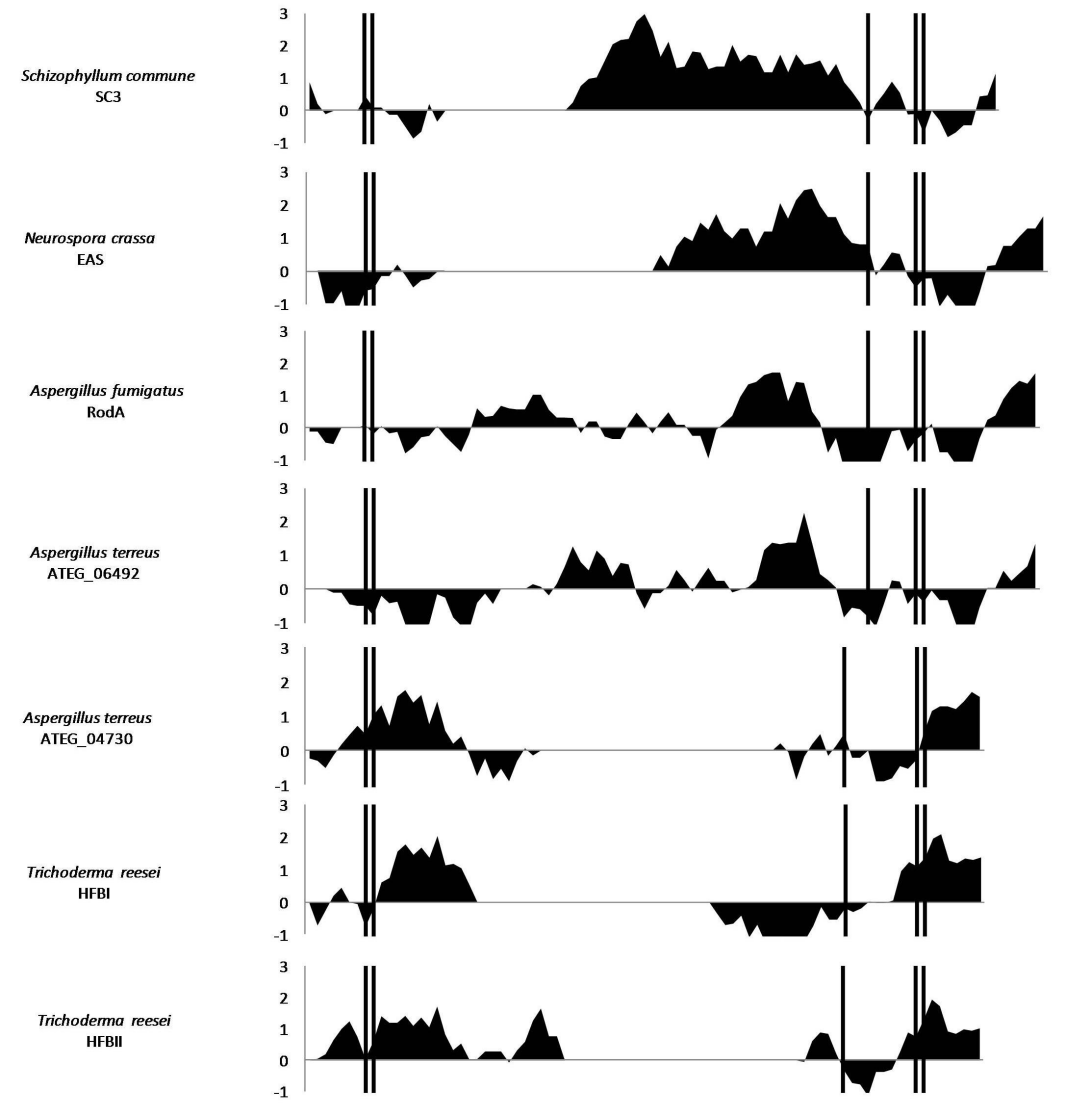
**Hydropathy patterns**. Hydropathy patterns of SC3 from *S. commune*, EAS from *N. crassa*, RodA from *A. fumigatus*, HFBI and HFBII from *T. reesei *and proteins ATEG_06492 and ATEG_04730 from *A. terreus*. The amino acids of the hydrophobins are shown along the x-axis, where cysteines are indicated by vertical lines. Hydrophobic amino acids are shown above the x-axis, while hydrophilic amino acids are shown below. Only the part of the sequence from the first to the eighth cysteine was used to create the hydropathy pattern.

**Figure 2:**
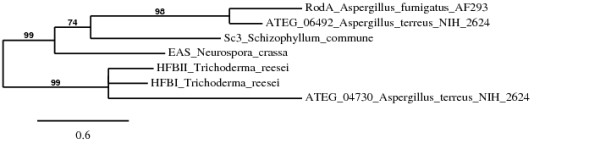
**Phylogenetic tree of class I and class II hydrophobins**. Sequences of SC3 (*S. commune*), EAS (*N. crassa*), RodA (*A. fumigatus*), HFBI and HFBII (*T. reesei*) were obtained from the National Center for Biotechnology Information (NCBI). The phylogenetic tree was constructed based on a multiple alignment of identified hydrophobins using Phylogeny.fr [22]. Branches with support values less than 50% were collapsed.

In *Aspergillus terreus *five different hydrophobins were identified. ATEG_06492 displayed a characteristic class I hydrophobin cysteine spacing pattern (CN{7}CCN{40}CN{16}CN{5}CCN{17}C), whereas a class II hydrophobin spacing pattern was observed for ATEG_04730 (CN{10}CCN{11}CN{16}CN{8}CCN{10}C). Comparison of ATEG_06492 and ATEG_04730 to hydropathy patterns of known class I and class II hydrophobins indicates that *A. terreus *has genes for both class I and class II hydrophobins (Figure 1). The hydrophobins SC3 (*Schizophyllum commune)*, EAS (*Neurospora crassa*) and RodA (*Aspergillus fumigatus*) are known class I hydrophobins, where the cysteine doublets are followed by a stretch of hydrophilic amino acids. Likewise, the cysteine doublets in ATEG_06492 are followed by a stretch of hydrophilic amino acids contrasting ATEG_04730, where hydrophobic amino acids follow the cysteine doublets. Similarly, the cysteine doublets are followed by hydrophobic amino acids in the known class II hydrophobins HFBI and HFBII from *Trichoderma reesei*. Therefore ATEG_06492 displays a characteristic class I hydropathy pattern, while ATEG_04730 displays a class II hydropathy pattern. Comparison of ATEG_04730 to class II hydrophobins HFBI and HFBII showed 37% and 35% sequence identity, while comparison to class I hydrophobins RodA, SC3 and EAS showed 21%, 16% and 20% sequence identity. In contrast ATEG_06492 showed 20% and 29% sequence identity to class II hydrophobins HFBI and HFBII, but 51%, 21% and 24% to class I hydrophobins RodA, SC3 and EAS. Furthermore, a phylogenetic analysis (Figure 2) revealed that ATEG_04730 clusters with HFBI and HFBI, while ATEG_06492 clusters with RodA, EAS and SC3, strongly indicating that ATEG_04730 can be classified as a class II hydrophobin, while ATEG_06492 is classified as a class I hydrophobin. As neither ATEG_06492 nor ATEG_04730 have physically been isolated or characterised, these can obviously only tentatively be classified as a class I and a class II hydrophobin respectively. This is the first report of an *Aspergillus *species with the potential to express both class I and class II hydrophobins.

## Conclusion

Analysis of nine genome sequences from seven Aspergilli revealed fifty hydrophobins, where each species displayed between two and eight hydrophobins. Twenty of the identified hydrophobins have not previously been described from these species. All identified hydrophobins contained two cysteine pairs, were approximately 100-200 AA in size, and displayed the common eight cysteine pattern. Besides the cysteines, very little amino acid sequence homology was observed. Twenty-three of the identified hydrophobins could be classified as class I hydrophobins based on their conserved cysteine spacing pattern and hydropathy pattern, but the majority seem to be intermediate forms. A single hydrophobin, ATEG_04730, from *Aspergillus terreus *displayed a clear class II cysteine spacing and had a class II hydropathy pattern. Furthermore, this hydrophobin grouped together with other known class II hydrophobins in a phylogenetic analysis, showing a close phylogenetic relationship to these. As *Aspergillus terreus *also has the potential to express a class I hydrophobin, this is the first reported case of an *Aspergillus *species with the potential to express both class I and class II hydrophobins.

## Methods

### Availability of genomic data

The sequences of *Aspergillus oryzae *RIB40, *Aspergillus niger *CBS 513.88, *Emericella nidulans *FGSC A4, *Aspergillus fumigatus *AF293, *Aspergillus fumigatus *A1163, *Aspergillus terreus *NIH 2624, *Aspergillus flavus *NRRL 3357 and *Aspergillus clavatus *NRRL 1 were obtained from the Central Aspergillus Data Repository (CADRE) [18], while the sequence of *Aspergillus niger *ATCC 1015 was obtained from DOE Joint Genome Institute.

### Identification of putative hydrophobins

A Perl program was constructed to search the nine *Aspergillus *genomes for putative hydrophobins by identification of the common C..CC..C..C..CC..C cysteine motif [2,3]. The identified putative hydrophobins were further sorted for size and number of cysteine residues resulting in fifty putative hydrophobins. The identified putative hydrophobin sequences were used to conduct a BLAST search against the NCBI (National Center for Biotechnology Information) non-redundant (nr) database to differentiate between known and newly identified hydrophobins. The sequences were examined for domains using Pfam to verify their function as hydrophobins [19] and the presence of and location of signal peptide cleavage sites using SignalP 3.0 to examine their theoretical ability to be secreted [20].

### Protein sequence analysis

A multiple sequence alignment of the identified hydrophobin sequences was conducted using MUSCLE [21] and based on this alignment a phylogenetic tree was constructed [22-25].

### Generation of hydropathy plots

Hydropathy patterns were determined using the hydropathy scale set by Kyte and Doolittle [26]. A nine amino acid window was used and data was extracted using Protscale on the ExPASy Proteomics Server [27]. The hydropathy patterns were aligned around the cysteine pairs placing gaps in the sequences where the hydrophobic and hydrophilic regions alternate. Only the part of the sequence from the first cysteine to the eight was used for examining the hydropathy pattern.

## Competing interests

The authors declare that they have no competing interests.

## Authors' contributions

BGJ carried out the sequence analysis, blast searches, domain searches, phylogenetic analysis, created hydropathy patterns and drafted the manuscript. MRA wrote the Perl program used for searching the *Aspergillus *sequences and participated in data analysis. MHP, JCF and IS gave general direction and manuscript revisions. All authors read and approved the final manuscript.

## Supplementary Material

Additional file 1 **Phylogenetic tree of identified hydrophobins in Aspergilli**. The phylogenetic tree was constructed based on a multiple alignment of identified hydrophobins using Phylogeny.fr (Dereeper *et al*., 2008). Branches with support values less than 50% were collapsed. N signifies any other amino acid than cysteine. Click here for file

Additional file 2 **Theoretical class of identified hydrophobins based on cysteine spacing and hydropathy plot**. The hydropathy plots were created using ProtScale (Gasteiger *et al*. 2005). Click here for file

Additional file 3 **Multiple alignment of putative hydrophobins in Aspergilli**. Comparison of hydrophobins identified in full genome sequenced Aspergilli. Amino acid residues are colored by conservation (> 80%). Figure created using Jalview [28]. Click here for file
