# Supplementary material for: Hydrophobins from Aspergillus species cannot be clearly divided into two classes
Source: BMC Res Notes. 2010 Dec 23;3:344. doi: 10.1186/1756-0500-3-344 (PMC3020181; doi:10.1186/1756-0500-3-344)
Supplement: Additional file 1 — Phylogenetic tree of identified hydrophobins in Aspergilli. The phylogenetic tree was constructed based on a multiple alignment of identified hydrophobins using Phylogeny.fr (Dereeper et al., 2008). Branches with support values less than 50% were collapsed. N signifies any other amino acid than cysteine. [file 1756-0500-3-344-S1.PDF]

Additional files

" Hydrophobins from *Aspergillus* species cannot be clearly divided into two classes"

B.G. Jensen, M.R. Andersen, M.H. Pedersen, J.C. Frisvad and I. Søndergaard

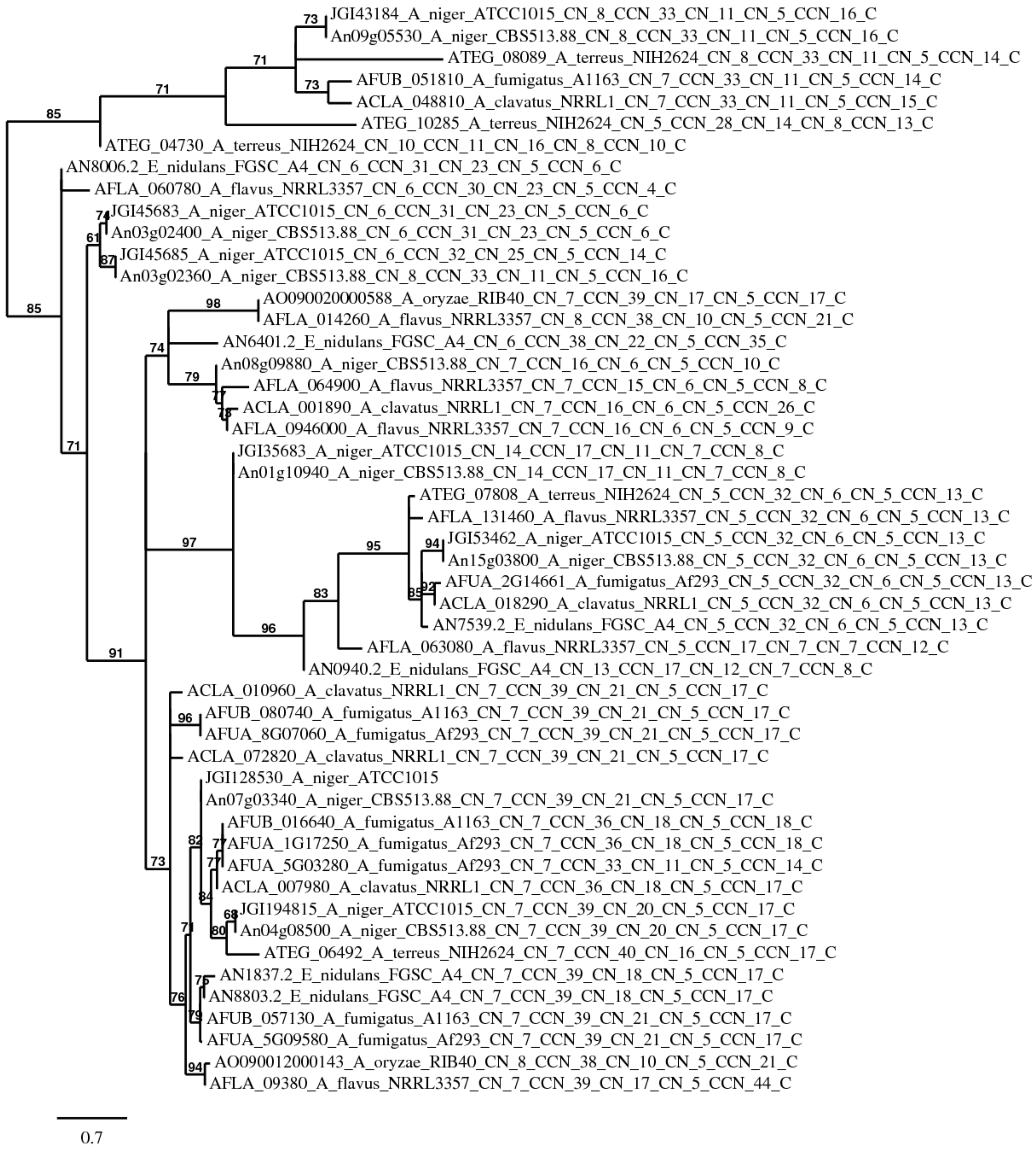

Additional file 1. Phylogenetic tree of identified hydrophobins in Aspergilli

The phylogenetic tree was constructed based on a multiple alignment of identified hydrophobins using Phylogeny.fr (Dereeper *et al.*, 2008). Branches with support values less than 50 % were collapsed. N signifies any other amino acid than cysteine.
