# Supplementary material for: Hydrophobins from Aspergillus species cannot be clearly divided into two classes
Source: BMC Res Notes. 2010 Dec 23;3:344. doi: 10.1186/1756-0500-3-344 (PMC3020181; doi:10.1186/1756-0500-3-344)
Supplement: Additional file 2 — Theoretical class of identified hydrophobins based on cysteine spacing and hydropathy plot. The hydropathy plots were created using ProtScale (Gasteiger et al. 2005). [file 1756-0500-3-344-S2.PDF]

Additional files

" Hydrophobins from *Aspergillus* species cannot be clearly divided into two classes"

B.G. Jensen, M.R. Andersen, M.H. Pedersen, J.C. Frisvad and I. Søndergaard

Species	Gene	Eight cysteine pattern	Class based on cysteine pattern	Class based on hydropathy plot	Theoretical class
A. oryzae RIB40	AO090012000143	CN{8}CCN{38}CN{10}CN{5}CCN{21}C	I	I	I
	AO090020000588	CN{7}CCN{39}CN{17}CN{5}CCN{17}C	I	I	I
A. niger CBS 513.88	An03g02360	CN{6}CCN{32}CN{25}CN{5}CCN{4}C	I	I	I
	An03g02400	CN{6}CCN{31}CN{23}CN{5}CCN{6}C	I	Intermediate	Intermediate
	An04g08500	CN{7}CCN{39}CN{20}CN{5}CCN{17}C	I	I	I
	An15g03800	CN{5}CCN{32}CN{6}CN{5}CCN{13}C	I	II	Intermediate
	An01g10940	CN{14}CCN{17}CN{11}CN{7}CCN{8}C	-	Intermediate	Intermediate
	An07g03340	CN{7}CCN{39}CN{21}CN{5}CCN{17}C	I	I	I
	An09g05530	CN{8}CCN{33}CN{11}CN{5}CCN{16}C	I	Intermediate	Intermediate
	An08g09880	CN{7}CCN{16}CN{6}CN{5}CCN{10}C	I	Intermediate	Intermediate
A. niger ATCC 1015	JGI128530	Fragment (similar to An07g03340)	I	Intermediate	Intermediate
	JGI35683	CN{14}CCN{17}CN{11}CN{7}CCN{8}C	-	Intermediate	Intermediate
	JGI45683	CN{6}CCN{31}CN{23}CN{5}CCN{6}C	I	Intermediate	Intermediate
	JGI45685	CN{6}CCN{32}CN{25}CN{5}CCN{14}C	I	I	I
	JGI53462	CN{5}CCN{32}CN{6}CN{5}CCN{13}C	I	II	Intermediate
	JGI194815	CN{7}CCN{39}CN{20}CN{5}CCN{17}C	I	I	I
	JGI43184	CN{8}CCN{33}CN{11}CN{5}CCN{16}C	I	Intermediate	Intermediate
E. nidulans FGSC A4	AN7539.2	CN{5}CCN{32}CN{6}CN{5}CCN{13}C	I	II	Intermediate
	AN8803.2	CN{7}CCN{39}CN{18}CN{5}CCN{17}C	I	I	I
	AN6401.2	CN{6}CCN{38}CN{22}CN{5}CCN{35}C	I	Intermediate	Intermediate
	AN8006.2	CN{6}CCN{31}CN{23}CN{5}CCN{6}C	I	I	I
	AN1837.2	CN{7}CCN{39}CN{18}CN{5}CCN{17}C	I	I	I
	AN0940.2	CN{13}CCN{17}CN{12}CN{7}CCN{8}C	-	II	Intermediate
A. fumigatus AF293	AFUA_8G07060	CN{7}CCN{39}CN{21}CN{5}CCN{17}C	I	I	I
	AFUA_5G09580	CN{7}CCN{39}CN{21}CN{5}CCN{17}C	I	I	I
	AFUA_2G14661	CN{5}CCN{32}CN{6}CN{5}CCN{13}C	I	II	Intermediate
	AFUA_1G17250	CN{7}CCN{36}CN{18}CN{5}CCN{18}C	I	I	I
	AFUA_5G03280	CN{7}CCN{33}CN{11}CN{5}CCN{14}C	I	I	I
A. fumigatus A1163	AFUB_016640	CN{7}CCN{36}CN{18}CN{5}CCN{18}C	I	I	I
	AFUB_057130	CN{7}CCN{39}CN{21}CN{5}CCN{17}C	I	I	I
	AFUB_080740	CN{7}CCN{39}CN{21}CN{5}CCN{17}C	I	I	I
	AFUB_051810	CN{7}CCN{33}CN{11}CN{5}CCN{14}C	I	II	Intermediate
A. terreus NIH 2624	ATEG_10285	CN{5}CCN{28}CN{14}CN{8}CCN{13}C	-	I	Intermediate
	ATEG_08089	CN{8}CCN{33}CN{11}CN{5}CCN{14}C	I	Intermediate	Intermediate
	ATEG_07808	CN{5}CCN{32}CN{6}CN{5}CCN{13}C	I	Intermediate	Intermediate
	ATEG_06492	CN{7}CCN{40}CN{16}CN{5}CCN{17}C	I	I	I
	ATEG_04730	CN{10}CCN{11}CN{16}CN{8}CCN{10}C	II	II	II
A. flavus NRRL 3357	AFLA_094600	CN{7}CCN{16}CN{6}CN{5}CCN{9}C	I	II	Intermediate
	AFLA_131460	CN{5}CCN{32}CN{6}CN{5}CCN{13}C	I	Intermediate	Intermediate
	AFLA_060780	CN{6}CCN{30}CN{23}CN{5}CCN{4}C	I	I	I
	AFLA_014260	CN{8}CCN{38}CN{10}CN{5}CCN{21}C	I	I	I
	AFLA_063080	CN{5}CCN{17}CN{7}CN{7}CCN{12}C	-	Intermediate	Intermediate
	AFLA_098380	CN{7}CCN{39}CN{17}CN{5}CCN{44}C	I	I	I
	AFLA_064900	CN{7}CCN{15}CN{6}CN{5}CCN{8}C	I	II	Intermediate
A. clavatus NRRL 1	ACLA_001890	CN{7}CCN{16}CN{6}CN{5}CCN{26}C	I	Intermediate	Intermediate
	ACLA_048810	CN{7}CCN{33}CN{11}CN{5}CCN{15}C	I	II	Intermediate
	ACLA_010960	CN{7}CCN{39}CN{21}CN{5}CCN{17}C	I	I	I
	ACLA_072820	CN{7}CCN{39}CN{21}CN{5}CCN{17}C	I	I	I
	ACLA_018290	CN{5}CCN{32}CN{6}CN{5}CCN{13}C	I	Intermediate	Intermediate
	ACLA_007980	CN{7}CCN{36}CN{18}CN{5}CCN{17}C	I	II	Intermediate

Additional file 2. Theoretical class of identified hydrophobins based on cysteine spacing and hydropathy plot.

The hydropathy plots were created using ProtScale (Gasteiger *et al.* 2005).
