# Supplementary material for: Hydrophobins from Aspergillus species cannot be clearly divided into two classes
Source: BMC Res Notes. 2010 Dec 23;3:344. doi: 10.1186/1756-0500-3-344 (PMC3020181; doi:10.1186/1756-0500-3-344)
Supplement: Additional file 3 — Multiple alignment of putative hydrophobins in Aspergilli. Comparison of hydrophobins identified in full genome sequenced Aspergilli. Amino acid residues are colored by conservation (> 80%). Figure created using Jalview [28]. [file 1756-0500-3-344-S3.PDF]

**Additional files**

" Hydrophobins from *Aspergillus* species cannot be clearly divided into two classes"

B.G. Jensen, M.R. Andersen, M.H. Pedersen, J.C. Frisvad and I. Søndergaard

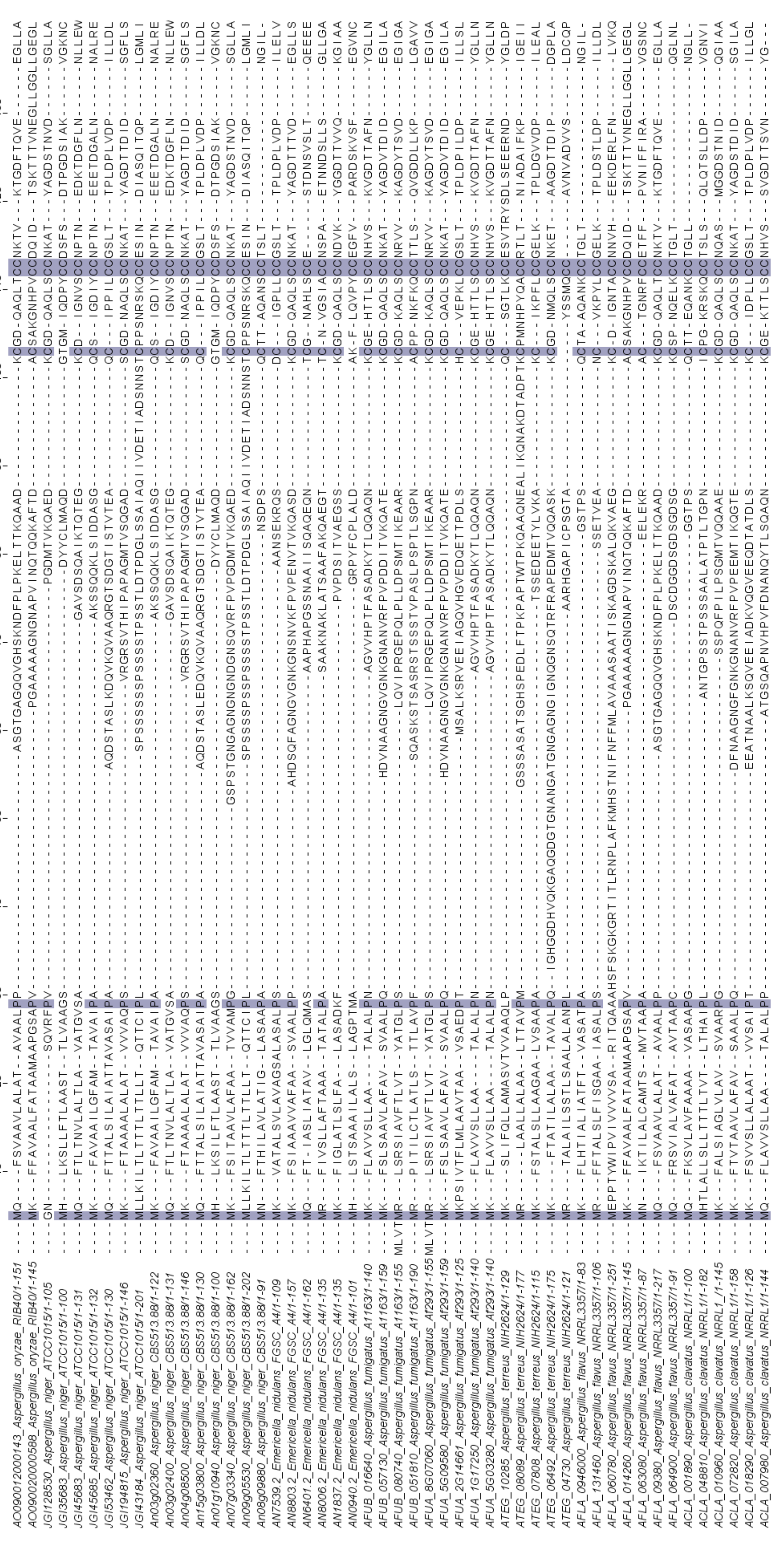

# Additional files

" Hydrophobins from *Aspergillus* species cannot be clearly divided into two classes"

B.G. Jensen, M.R. Andersen, M.H. Pedersen, J.C. Frisvad and I. Søndergaard

```
                                                       150       160       170       180       190       200       210       220       230       240       250       260       270
AO090012000143_Aspergillus_oryzae_RIB40/1-151        G-LLSN--LLGA-GQGSQG-LGLLDECTNIPVIPIISIASPQ-----EKCKQPISCCQNT--KSSADGDL------------------VGIGLP-CIALGSLL-------------------------------------
AO090020000588_Aspergillus_oryzae_RIB40/1-145        GGVLNN--LVGGEPGACSGLVSALN----------------------KQCQTSIGCCQQNAKGDNYQSGL------------------LNLNLQAPCLLSNGL-------------------------------------
JGI128530_Aspergillus_niger_ATCC1015/1-105           G-TLSN--LIGT-GSGSEG-LGLFQECSKLPIQ-IPIIGIAVQDIISKQCQQNIACCQSS--PS----------------------------------------------------------------------------
JGI35683_Aspergillus_niger_ATCC1015/1-100            Q-SMDG--LEWT-DQCPQG--------------------------------GTVKCCYT-----------------------------IGPQFICTAEAEENTDDD----------------------------------
JGI45683_Aspergillus_niger_ATCC1015/1-131            G-VIGS--LVNGQGSACAP-ISLIDELGILALVKDTPDG--------PVCENVIACCPGQ--------------------------------GAQCVAIGDGSGSGSGYSD-----------------------------
JGI45685_Aspergillus_niger_ATCC1015/1-132            GLLIGS--LVNGKGSACAP-TSLIGS-LNLLAFFEEGEDDDK-----SYCKNVIACCPGG--NLAD----------------------LEFRKEQCAALDGY--------------------------------------
JGI53462_Aspergillus_niger_ATCC1015/1-130            GIDASN--IVGSIGLLCKDYD--------------------------DSCTSAPCCCTEI--NLL-----------------------NGLVALGCSSLEQ---------------------------------------
JGI194815_Aspergillus_niger_ATCC1015/1-146           G-VLSN--LLGA-GSGSQG-IGLADECSPLDLQALDLVGLQNLLN--DQCEQTAACCQGS--GSDTEGNL------------------IGVGLP-CLALGSLL-------------------------------------
JGI43184_Aspergillus_niger_ATCC1015/1-201            P-WIGGIDISSVLGLDCTGMSDDTPN---------------------DQCSHQVMCCDGQ--PNSEGS--------------------SQPISSACKPFDDAIKDKNDAINDSKQRESMLVAQASASASASASASASA
An03g02360_Aspergillus_niger_CBS513.88/1-122         GLLIGS--LVNGKGSACAP-TSLIGS-LNLLAFFEEGEDDDK-----SYCKNVIACCPGG----------------------------------NCAALDGY--------------------------------------
An03g02400_Aspergillus_niger_CBS513.88/1-131         G-VIGS--LVNGQGSACAP-ISLIDELGILALVKDTPDG--------PVCENVIACCPGQ--------------------------------GAQCVAIGDGSGSGSGYSD-----------------------------
An04g08500_Aspergillus_niger_CBS513.88/1-146         G-VLSN--LLGA-GSGSQG-IGLADECSPLDLQALDLVGLQNLLN--DQCEQTAACCQGS--GSDTEGNL------------------IGVGLP-CLALGSLL-------------------------------------
An15g03800_Aspergillus_niger_CBS513.88/1-130         GIDASN--IVGSIGLLCKDYD--------------------------DSCTSAPCCCTEI--NLL-----------------------NGLVALGCSSLEQ---------------------------------------
An01g10940_Aspergillus_niger_CBS513.88/1-100         Q-SMDG--LEWT-DQCPQG--------------------------------GTVKCCYT-----------------------------IGPQFICTAEAEENTDDD----------------------------------
An07g03340_Aspergillus_niger_CBS513.88/1-162         G-TLSN--LIGT-GSGSEG-LGLFQECSKLPIQ-IPIIGIAVQDIISKQCQQNIACCQSS--PSTTDDDL------------------IGLGLP-CIALGSLV-------------------------------------
An09g05530_Aspergillus_niger_CBS513.88/1-202         P-WIGGVDISSVLGLDCTGMSDDTPN---------------------DQCSHQVMCCDGQ--PNSEGS--------------------SQPISSACKPFDDAIKDKNDAINDSKQRESMLVAQASASASASASASASA
An08g09880_Aspergillus_niger_CBS513.88/1-91          --------NVNVLPALCVPLV--------------------------GNCNNQAACCETNGIPDTHG---------------------CSQGLLNCLTVQV---------------------------------------
AN7539.2_Emericella_nidulans_FGSC_A4/1-109           GIDAAA--IVGSVGLLCHAYD--------------------------DTCTSEPCCCTEA--NLL-----------------------GGTLGLGCGPLEHGH-------------------------------------
AN8803.2_Emericella_nidulans_FGSC_A4/1-157           G-ALSG--LIGA-GSGAEG-LGLFDQCSKLDVAVLIGIQDLVN----QKCKQNIACCQNS--PSSADGNL------------------IGVGLP-CVALGSIL-------------------------------------
AN6401.2_Emericella_nidulans_FGSC_A4/1-162           G-LLHL--LGGTSSVLSDGLLGKYSGCSSLASVEGILGAGGNQGLVSGQCNNHVACCDAG--DNELVGFLEPGVAVLKPNSDGIQNGLANVAVP-CVPVQVL--------------------------------------
AN8006.2_Emericella_nidulans_FGSC_A4/1-135           G-LLNG--LSGNTGSACAK-ASLIDQ-LGLLALVDHTEEG-------PVCKNIVACCPEG--T------------------------------TNCVAVDNAGAGTKAE-------------------------------
AN1837.2_Emericella_nidulans_FGSC_A4/1-135           G-LLSD--LLGA-GSAAEG-LGAFSGCSKLDISLLIGIEDILN----QKCKQNIACCAKS--GGSADGDL------------------VGATLP-CIALGSIL-------------------------------------
AN0940.2_Emericella_nidulans_FGSC_A4/1-101           I-DVSS--DEDFVKTCPKG--------------------------------GTPKCCYS-----------------------------IGPKVICTTEVGDGVDK-----------------------------------
AFUB_016640_Aspergillus_fumigatus_A1163/1-140        G-------LLGNAISGPEG-VGILSGCQKISVTALIGVDDLLN----KQCQQNVACCQDN--KSVATGGL------------------INIATPACVALDSII-------------------------------------
AFUB_057130_Aspergillus_fumigatus_A1163/1-159        G-TLKN--LIGG-GSGTEG-LGLFNQCSKLDLQ-IPIIGIPIQDLVNQKCKQNIACCQNS--PSDASGSL------------------IGLGLP-CIALGSIL-------------------------------------
AFUB_080740_Aspergillus_fumigatus_A1163/1-155        G-LLSN--LAGG-GSGISS-ILAFDQCSRLDAQ-VPVVLLPIQDLLNQHCKQNVACCQKN--PGDASSSG------------------VGVSLP-CIALGSVA-------------------------------------
AFUB_051810_Aspergillus_fumigatus_A1163/1-190        P-LVGAIQVNSLVGVSCRPMADAAPE---------------------STCGNAVMCCDSSTVGG------------------------DDLMQTSCQDFALAKKREREAIERQQRRFSEYQRMMLSQSATPAPTSTGVDV
AFUA_8G07060_Aspergillus_fumigatus_Af293/1-155       G-LLSN--LAGG-GSGISS-ILAFDQCSRLDAQ-VPVVLLPIQDLLNQHCKQNVACCQKN--PGDASSSG------------------VGVSLP-CIALGSVA-------------------------------------
AFUA_5G09580_Aspergillus_fumigatus_Af293/1-159       G-TLKN--LIGG-GSGTEG-LGLFNQCSKLDLQ-IPIIGIPIQDLVNQKCKQNIACCQNS--PSDASGSL------------------IGLGLP-CIALGSIL-------------------------------------
AFUA_2G14661_Aspergillus_fumigatus_Af293/1-125       GINAAQ--IVGSVGLLCHPWT--------------------------EECSSAPCCCTEA--NLL-----------------------GGTLALGCSKL-----------------------------------------
AFUA_1G17250_Aspergillus_fumigatus_Af293/1-140       G-------LLGNAISGPEG-VGILSGCQKISVTALIGVDDLLN----KQCQQNVACCQDN--KSVATGGL------------------INIATPACVALDSII-------------------------------------
AFUA_5G03280_Aspergillus_fumigatus_Af293/1-140       G-------LLGNAISGPEG-VGILSGCQKISVTALIGVDDLLN----KQCQQNVACCQDN--KSVATGGL------------------INIATPACVALDSII-------------------------------------
ATEG_10285_Aspergillus_terreus_NIH2624/1-129         D-LNDA--NICDNGSLVED-EFDLAIC--------------------DSVDHVPCCCLAFVPTRALARTQCDRTTDED----------TGPKAPGSYGIDTDLTPQLLSKD-----------------------------
ATEG_08089_Aspergillus_terreus_NIH2624/1-177         P-QLKGANLASPMSFECKPMTPGDPP---------------------EKCAHQASCCGSD--PKEA----------------------SAIDINSCTDYREEMNKTIEALERNKKMHSRAVVLPVYV-------------
ATEG_07808_Aspergillus_terreus_NIH2624/1-115         HLGPEA--IVGSIGLACHA--YD------------------------ETCTTAPKCCSEA--NLL-----------------------DGTVALGCNDLANPADAL----------------------------------
ATEG_06492_Aspergillus_terreus_NIH2624/1-175         D-ALKG--VLGGAGSGSEG-LGLLEQCGKLDI--IPIISDLIN----KHCDAKAACCQDS--GSSADNSL------------------IGLNLP-CIALTSLI-------------------------------------
ATEG_04730_Aspergillus_terreus_NIH2624/1-121         PGPLPS--SAAEFKEICAK------------------------------IGDKPCCCTLA----------------------------LAGQAIACQNPAGMDEDSGSSESESDSGSGGGGGDA----------------
AFLA_0946000_Aspergillus_flavus_NRRL3357/1-83        --------NLNVLPALCLPLV--------------------------GSCNNQAACCETN-----------------------------GIGLLNCLTVQV---------------------------------------
AFLA_131460_Aspergillus_flavus_NRRL3357/1-106        GIDAAS--IVGSVGLLCHAWD--------------------------ETCETGPKCCTEA--NLL-----------------------GGTVALGCSDLATED-------------------------------------
AFLA_060780_Aspergillus_flavus_NRRL3357/1-251        G-LIDI--LAGNEDYACAK-SGVIDE-WNLFSLVKQTNDG-------PVCKNVTACCSSG----------------------------------KCVAIDGSAEKKKESDKEDDKEDDKEDDREDDKEGDNEGDNEGDDK
AFLA_014260_Aspergillus_flavus_NRRL3357/1-145        GGVLNN--LVGGEPGACSGLVSALN----------------------KQCQTSIGCCQQNAKGDNYQSGL------------------LNLNLQAPCLLSNGL-------------------------------------
AFLA_063080_Aspergillus_flavus_NRRL3357/1-87         --------IAQKTGNCPTG--------------------------------TTEHCCTSNLP--------------------------LRNGDLACTQL-----------------------------------------
AFLA_09380_Aspergillus_flavus_NRRL3357/1-217         G-LLSN--LLGA-GQGSQG-LGLLDECTNIPVIPIIPIASPQ-----EQCKQPISCCQNTKSSAVSRINAQYTQLSLTITGWRPRRYWSSLHRSRLSPVSDCIREIGSSRSTGWKMAMKCFHSVLFCVLYWLLVSWNPRS
AFLA_064900_Aspergillus_flavus_NRRL3357/1-91         G-ILPA---------LCLPLL--------------------------ANCNNQAACCEAK------------------------------GGLLNCLTIQL---------------------------------------
ACLA_001890_Aspergillus_clavatus_NRRL1/1-100         --------NINILPALCLPLV--------------------------GTCNNQAACCESN--GIVSRNRIIGHQTADE----------ESKGLLNCLTIQI---------------------------------------
ACLA_048810_Aspergillus_clavatus_NRRL1/1-182         P-LLSGMQLTSLIGLSCRTMSDEHTK---------------------TDCADALMCCDAA--GEVGA---------------------SNILQTKCEEFDLAKAKEIAAVERAQGQPSSAARMFGASSSSVVLGSGSGSG
ACLA_010960_Aspergillus_clavatus_NRRL1_/1-145        G-LLKD--VLGG-GSVNQG-VNLFHQCAKLDLQ-IPIIGVPIQDALNQRCKQNVACCQAP--KADASNDG------------------VALSLP-CIALGSVI-------------------------------------
ACLA_072820_Aspergillus_clavatus_NRRL1/1-158         G-TLKN--LIGG-GSGHQG-LGLFDQCSKLDLQ-IPIIGIPIQDLINQQCKQNIACCQKS--TADASGSL------------------IGLGLP-CVALGSIL-------------------------------------
ACLA_018290_Aspergillus_clavatus_NRRL1/1-126         GINAAE--IVGSIGLLCHAWT--------------------------PECSSAPCCCTEA--NLL-----------------------GGAVALGCSKL-----------------------------------------
ACLA_007980_Aspergillus_clavatus_NRRL1/1-144         --LLNG--LLGNTLSGPEG-VGLLSGCTKLDVTALIGVNDLLN----KQCQQNVACCQGT--ESTATGGL------------------VNLALP-CVALGSII-------------------------------------
```

**Additional files**

" Hydrophobins from *Aspergillus* species cannot be clearly divided into two classes"

B.G. Jensen, M.R. Andersen, M.H. Pedersen, J.C. Frisvad and I. Søndergaard

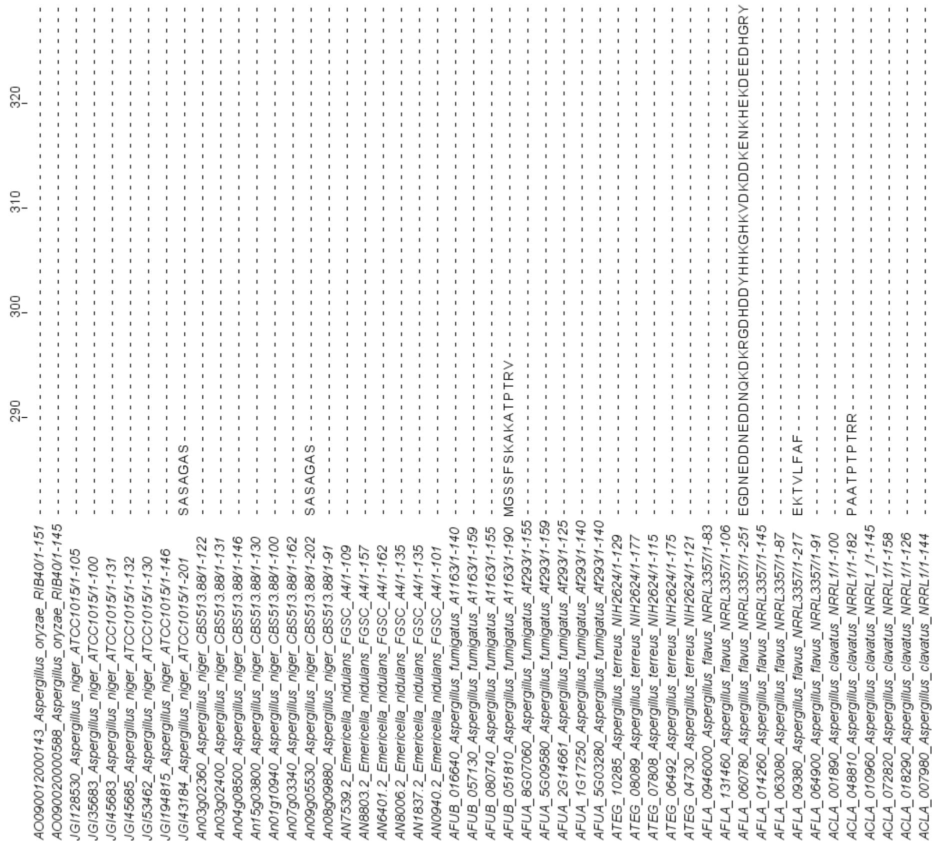

**Additional file 3. Multiple alignment of putative hydrophobins in Aspergilli**

Comparison of hydrophobins identified in full genome sequenced Aspergilli. Amino acid residues are colored by conservation (>80 %). Figure created using Jalview (Waterhouse *et al.* 2009)
